# Cementitious Composites with Hybrid UHMWPE and CF/PP Fiber: A Study on Compressive, Tensile, Flexural and Impact Performance

**DOI:** 10.3390/ma19102131

**Published:** 2026-05-19

**Authors:** Lihui Yang, Zhen Yang, Xiong Xing

**Affiliations:** School of Civil Engineering, Beijing Jiaotong University, Beijing 100044, China; yz_bjtu2023@163.com (Z.Y.); xx1023555@163.com (X.X.)

**Keywords:** hybrid fiber, impact resistance, Weibull distribution model, elastic modulus, static and dynamic strength, damage mechanics

## Abstract

Ultra-high molecular weight polyethylene (UHMWPE) fibers have recently emerged as a promising reinforcement material in fiber-reinforced concrete (FRC). To investigate the synergistic effects and reinforcing mechanisms of fibers with different elastic moduli within the concrete matrix, a series of hybrid fiber-reinforced concrete (HFRC) specimens were prepared by incorporating 0.25 vol%, 0.5 vol%, and 0.75 vol% carbon fibers (CFs) or polypropylene (PP) fibers into concrete containing 1 vol% UHMWPE fibers. The mechanical performance of the prepared composites was systematically evaluated through compressive, splitting tensile, flexural, and drop-weight impact tests. The experimental results indicate that concrete reinforced solely with UHMWPE fibers exhibits higher compressive strength but lower tensile strength, flexural strength, ductility, and impact toughness than the hybrid fiber systems. For both UHMWPE-CF and UHMWPE-PP hybrid concretes, the initial cracking impact resistance and failure impact resistance increased progressively with increasing CF or PP content. At equivalent fiber volume fractions, UHMWPE-PP hybrid concrete demonstrated superior resistance to initial cracking, whereas UHMWPE-CF hybrid concrete exhibited better post-failure impact resistance. Furthermore, fractal theory was employed to quantitatively characterize the impact damage behavior of HFRC specimens. The impact damage evolution equation is established by using the two-parameter Weibull distribution model. The findings provide theoretical and experimental support for the design and optimization of hybrid fiber-reinforced concrete subjected to impact loading.

## 1. Introduction

As the most widely used construction material globally, concrete demand has been continuously rising and is widely favored in the market [1]. However, its high self-weight, insufficient tensile strength, and usual working state with cracks lead to brittle failure when the material fractures, which significantly affects the safety of its application. In engineering structure design, not only quasi-static loads but also dynamic loads caused by explosions, impacts, etc., need to be fully considered. Ordinary concrete has limited ductility and exhibits brittle failure characteristics when subjected to impact loads [2], which restricts its application in seismic and anti-explosion design. To improve the mechanical behavior of concrete under dynamic loads, researchers innovatively incorporate synthetic or natural fiber materials into traditional concrete formulations to construct fiber-reinforced concrete (FRC). This measure significantly improves the mechanical properties of concrete; with the addition of fibers, the concrete structure is improved, showing better toughness and durability when subjected to impact, effectively delaying the crack propagation rate and enhancing the overall bearing capacity [3].

Currently, commonly used fibers include high elastic modulus fiber materials, such as steel fibers (SFs) [4], carbon fibers (CFs) [5], basalt fibers (BFs) [6], and polyethylene fibers (PEs). Low elastic modulus fiber materials include polyvinyl alcohol (PVA) fibers [7] and polypropylene (PP) fibers [8], etc. When fibers are uniformly distributed in the concrete matrix, they can prevent the generation and propagation of cracks through the bridging effect or other mechanisms, thereby significantly improving the mechanical properties of concrete [9]. However, each fiber material has corresponding advantages and disadvantages; therefore, the comprehensive performance of fiber concrete mixed with only a single type of fiber is often limited. According to composite material theory, mixing fibers with different mechanical properties into concrete can give full play to the advantages of each fiber, thereby obtaining composite materials with superior performance. Therefore, scholars at home and abroad have focused their research on the impact resistance of hybrid fiber-reinforced concrete (HFRC).

Research on single-fiber-reinforced concrete has provided important references for the mix ratio optimization of hybrid fiber systems. Luo et al. [10] and P.P. Li et al. [11] explored the impact resistance of concrete with different aggregates and single-fiber-reinforced concrete through different impact test devices. Yu et al. [12] and Perumal et al. [13] respectively studied the influence of single-fiber content (such as basalt fiber and steel fiber) on the impact resistance of concrete. These studies clarified the reinforcement mechanism and limitations of single fibers, providing theoretical and experimental support for the selection, content control, and synergistic effect of hybrid fibers.

Early research on HFRC mainly focused on the hybrid system of mineral admixtures and fibers. Sadi I. Haruna [14] and Dhale. et al. [15] used the drop-weight impact test method to study the effect of mixed addition of fine nano silica (NS) and fine steel fibers (SFs) on the impact resistance of concrete. The results showed that the synergistic effect of fine SF and NS can significantly improve the impact resistance of concrete, but when the content of SF and NS is too high, the mechanical properties of the specimens deteriorate. With in-depth research, scholars have shifted their focus to the hybrid effect of different types of fibers. Wang et al. [16] studied the hybrid effect of steel wire steel fibers, milled steel fibers, carbon fibers, and polypropylene fibers, and found that the incorporation of hybrid fibers can significantly improve the impact resistance of concrete. Among them, carbon fibers have the most prominent reinforcement effect; compared with ordinary concrete, the impact resistance of hybrid fiber concrete is increased by 8 times, confirming the superiority of hybrid fibers. For the hybrid system of aramid fibers (AFs) and carbon fibers (CFs), Yeou-Fong Li et al. [17] explored the effect of fibers with different mix ratios on the mechanical properties and impact resistance of concrete based on a total mass content of 1%. Meanwhile, scanning electron microscopy (SEM) and energy dispersive spectroscopy (EDS) were used to analyze the regulatory effect of coupling agents on the bonding performance between fibers and the matrix. The study confirmed that when the content ratio of AF to CF is 1:1, the compressive strength, tensile strength, and energy absorption effect of concrete reach the optimal level [18].

Among numerous hybrid systems, the hybrid system of steel fibers (SFs) and carbon fibers (CFs) has become a research hotspot in the impact resistance of HFRC due to its simultaneous consideration of the impact strength and toughness of concrete. Weimin Song et al. [19] explored the effect of hybrid incorporation of steel fibers and carbon fibers on the impact resistance and compressive strength of concrete through drop-weight impact tests and compressive tests. The results showed that the hybrid incorporation of the two fibers can simultaneously improve the compressive strength and impact toughness of concrete, and with the increase in steel fiber volume content, the initial cracking impact times of the specimens gradually increase, and the impact resistance is significantly enhanced. Dong-Ju Seo et al. [20] compared the impact resistance of steel–carbon fiber hybrid concrete with concrete mixed with only steel fibers or carbon fibers through a self-made drop-weight impact test device, and found that the hybrid fiber system can give full play to the bridging effect, and its improvement effect on impact resistance and toughness is better than that of single-fiber-reinforced concrete.

The above studies show that HFRC generally has better impact resistance than single-fiber-reinforced concrete. However, most existing studies have focused on hybrid systems that combine high-modulus fibers with low-modulus fibers, while direct comparisons between hybrid systems composed of different types of high-modulus fibers remain limited. In particular, the different roles of CFs and PP fibers in UHMWPE-based hybrid concrete under impact loading have not yet been fully clarified. Therefore, this study investigates UHMWPE/PP and UHMWPE/CF hybrid systems incorporated into C30 concrete and systematically evaluates their static mechanical properties, drop-weight impact resistance, and damage evolution behavior. In addition, a Weibull-based statistical model is established to preliminarily describe the damage characteristics of HFRC.

## 2. Materials and Methods

### 2.1. Materials

**Figure 1:**
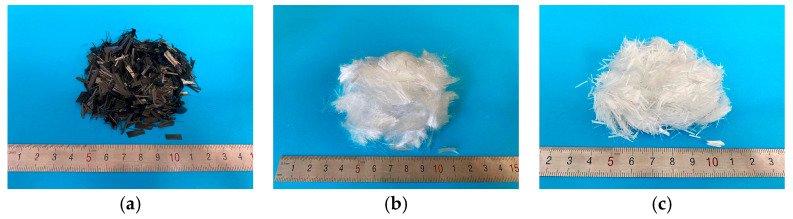
Images of fibers used in this study. (**a**) Carbon fiber; (**b**) UHMWPE fiber; (**c**) polypropylene fiber.

The materials used in this study included P.O 42.5 Portland cement, natural river sand, well-graded crushed stone with a particle size range of 5–25 mm, and a polycarboxylate-based high-range water-reducing admixture. Three types of fibers were used: ultra-high-molecular-weight polyethylene (UHMWPE) fiber, carbon fiber, and polypropylene (PP) fiber, as shown in Figure 1. The fibers were tested by the manufacturer in accordance with the relevant national standards before delivery.

The physical properties of the fibers used in this study are presented in Table 1. The carbon fiber had a length of 10 mm and a diameter of 7 μm with tensile strength and elastic modulus of 4900 MPa and 230 GPa, respectively. The ultra-high-molecular-weight polyethylene (UHMWPE) fiber had a length of 9 mm and a diameter of 25 μm with tensile strength and elastic modulus of 3100 MPa and 122 GPa respectively. The polypropylene (PP) fiber had a length of 9 mm and a diameter of 18 μm with tensile strength and elastic modulus of 500 MPa and 3.9 GPa respectively.

### 2.2. Mixing Ratios and Specimen Production

**Figure 2:**
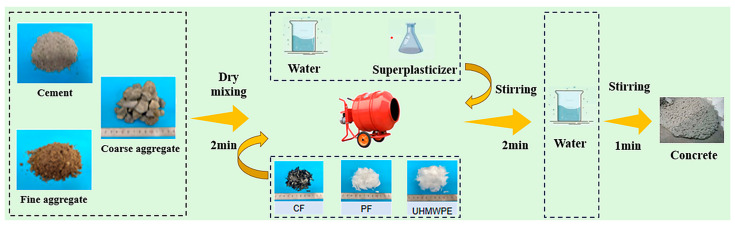
Schematic diagram of concrete mixing process.

The concrete mix proportions and specimen design are presented in Table 2. A total of 10 groups of cement-based mixtures with different fiber contents were prepared in this study. The water–cement ratio (W/C) was fixed at 0.43. To ensure adequate workability during mixing, a polycarboxylate superplasticizer accounting for 0.6% of the cement mass was added. For specimen preparation, cement, river sand, and crushed stone were first dry-mixed for 2 min to ensure uniform blending. Then, one-third of the mixing water was added, and wet mixing was carried out for 1 min before fiber incorporation. To minimize fiber agglomeration, fibers were added in batches and dry-mixed for another 1 min. Subsequently, the remaining water and superplasticizer were introduced, and mixing continued for 1 min. The fresh mixture was cast into oil-coated molds and compacted on a vibration table, while manual rodding was performed simultaneously to remove entrapped air bubbles. The top surface was leveled and smoothed, and the specimens were covered with plastic wrap to prevent moisture evaporation. All specimens were cured in a chamber at 20 ± 2 °C and a relative humidity of 95 ± 5% for 24 h before demolding and then standard-cured under the same conditions for 28 days. The mixing procedure is illustrated in Figure 2.

In this study, cube compressive strength tests, splitting tensile strength tests, four-point bending flexural tests, and drop-weight impact tests were conducted. Standard cubic specimens with a side length of 150 mm were used for the compressive, splitting tensile, and drop-weight impact tests. Prismatic specimens with dimensions of 400 mm × 100 mm × 100 mm were prepared for the four-point bending test. A total of 90 cubic specimens and 30 prismatic specimens were fabricated, with three specimens in each group.

### 2.3. Test Methods

#### 2.3.1. Axial Compression Test

**Figure 3:**
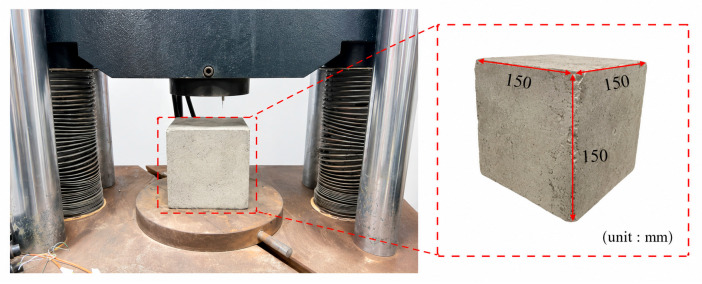
Axial compressive test setup.

The axial compression test setup is shown in Figure 3. All tests were performed in accordance with the Standard for Test Methods of Physical and Mechanical Properties of Concrete (GB/T 50081-2019) [21]. The concrete grade used in this study was C30. The compressive strength of fiber-reinforced concrete specimens after 28 days of curing was measured using a universal testing machine. The loading rate was controlled at 0.5 MPa/s with continuous and uniform loading. The failure load was recorded when the specimen failed, and the cube compressive strength was calculated according to Equation (1).

(1) $$f_{cu}=\frac{F}{A}$$

where *f_cu_* is the cube compressive strength of concrete (MPa), *F* is the failure load of the specimen (N), and *A* is the bearing area of the specimen (mm^2^).

#### 2.3.2. Splitting Tensile Test

**Figure 4:**
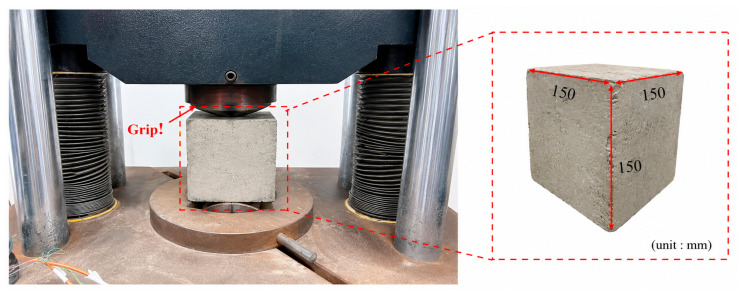
Splitting tensile test setup.

The test setup for splitting tensile strength is illustrated in Figure 4. All tests were conducted in accordance with the Standard for Test Methods of Physical and Mechanical Properties of Concrete (GB/T 50081-2019) [21]. The concrete grade adopted was C30. The splitting tensile strength of fiber-reinforced concrete (FRC) specimens after 28 days of curing was measured using a universal testing machine. The loading rate was controlled at 0.05 MPa/s with continuous and uniform loading. The failure load was recorded at specimen failure, and the splitting tensile strength was calculated according to Equation (2).

(2) $$f_{ts}=\frac{2F}{\pi A}=0.637\frac{F}{A}$$

where *f_ts_* is the splitting tensile strength of concrete (MPa), *F* is the failure load of the specimen (N), and *A* is the bearing area of the specimen (mm^2^).

#### 2.3.3. Flexural Performance Test

**Figure 5:**
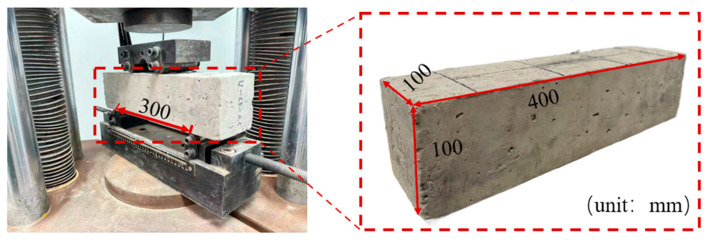
Flexural performance test setup.

The test setup for the four-point bending test is illustrated in Figure 5. Prismatic specimens with dimensions of 100 mm × 100 mm × 400 mm were prepared for the flexural performance test. All tests were carried out in accordance with the Standard for Test Methods of Physical and Mechanical Properties of Concrete (GB/T 50081-2019) [21], and the concrete grade was C30. The flexural strength of fiber-reinforced concrete specimens after 28 days of curing was measured using a universal testing machine. The loading rate was controlled at 0.05 MPa/s with continuous and uniform loading. The failure load was recorded at specimen failure, and the flexural strength was calculated according to Equation (3). Since non-standard specimens were adopted, a size conversion factor of 0.85 was applied to obtain the actual flexural strength.

(3) $$f_{f}=0.85\frac{FL}{bh^{2}}$$

where *f_f_* is the flexural strength of concrete (MPa), *F* is the failure load of the specimen (N), *L* is the span between supports (mm), *b* is the width of the specimen (mm), and *h*^2^ is the height of the specimen (mm).

#### 2.3.4. Impact Resistance Test

**Figure 6:**
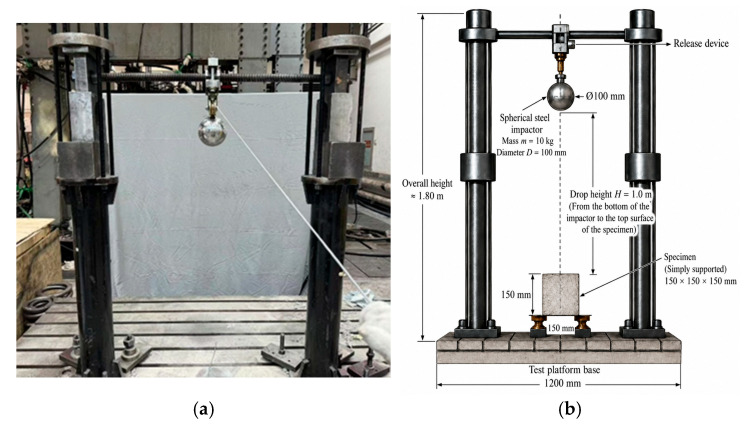
Drop-weight impact test setup. (**a**) Impact device. (**b**) Schematic diagram of device.

The drop-weight impact test was performed in accordance with the ACI 544 recommended method, and the test setup is illustrated in Figure 6. In compliance with CECS 13:2009 [22], a spherical steel impactor with a mass of 10 kg and a diameter of 10 cm was released freely from a height of 1 m above the specimen, and the impact point was aligned with the center of the upper surface of the specimen. During the test, the specimen was simply supported on the base of the testing platform, and no additional confinement or restraint was applied. The contact between the impactor and the specimen was a direct steel–concrete contact under free-fall loading. After each impact, the specimen surface was visually examined and photographed to record crack initiation and propagation. The number of impacts corresponding to the first crack initiation *N*_1_ the number of impacts at final failure *N*_2_ and the failure mode of the specimen were recorded. Notably, the specimen is considered to have failed when the displacement of any three faces of the specimen exceeds 10 mm. The impact energy absorption *W*, post-cracking energy absorption Δ*W*, and impact ductility ratio *β* were calculated according to Equations (4)–(7).

(4) $$W_{1}=N_{1}mgh$$

(5) $$W_{2}=N_{2}mgh$$

(6) $$\Delta W=W_{2}-W_{1}$$

(7) $$\beta =(N_{2}-N_{1})/N_{1}$$

where *N*_1_ is the number of impacts at initial cracking, *N*_2_ is the number of impacts at failure, *W*_1_ is the impact energy absorption at initial cracking (J), *W*_2_ is the impact energy absorption at failure (J), *g* is the acceleration due to gravity taken as 9.8 m/s^2^, *m* is the mass of the impact ball taken as 10 kg, *h* is the drop height of the impact ball taken as 1 m, Δ*W* is the post-cracking energy absorption (J), and *β* is the ductility ratio.

#### 2.3.5. Fractal Dimension

**Figure 7:**
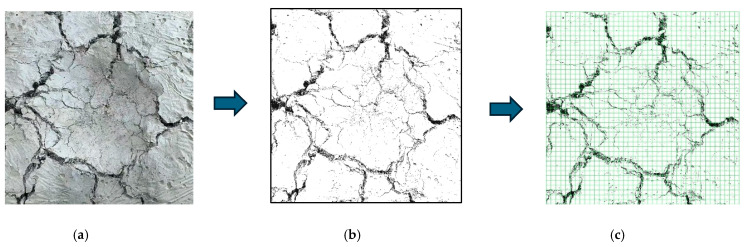
Image processing procedure of U-CF0.75. (**a**) Original image; (**b**) grayscale processing; (**c**) grid division.

**Figure 8:**
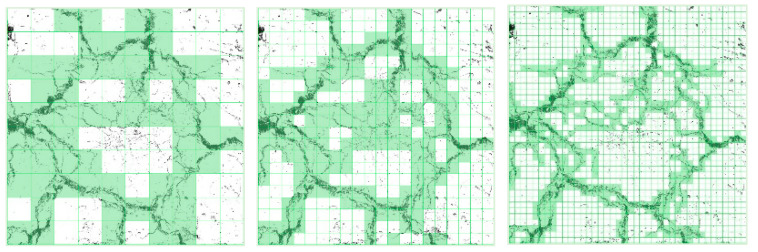
Coverage grid with different *r.*

**Figure 9:**
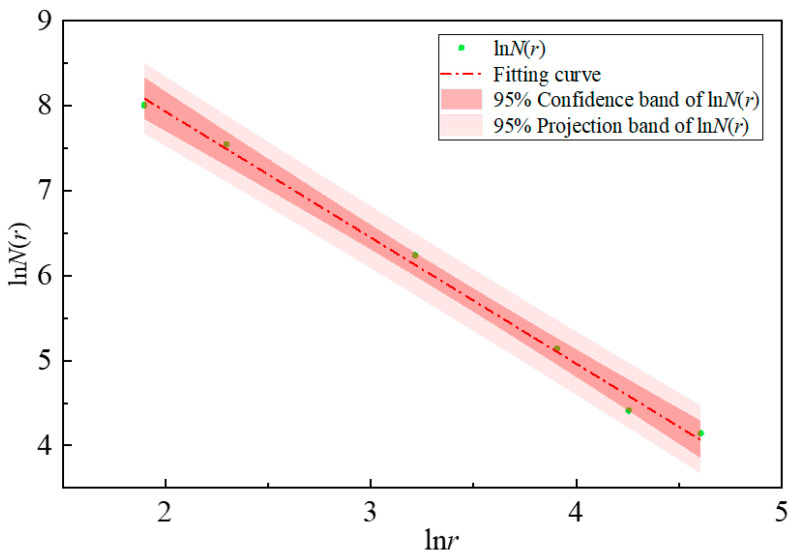
Fitting curve of U-CF0.75.

Judging the damage degree based on the crack width, length, quantity, and other failure characteristics often relies on the knowledge and experience of researchers. Cracks in fiber-reinforced concrete (FRC) under failure typically exhibit irregularity, discontinuity, and self-similarity [23]. Therefore, fractal theory was introduced to quantitatively analyze the impact damage degree of fiber-reinforced concrete under impact loading using the box-counting method. In this study, the fractal dimension *D_f_* of crack-containing images was calculated as a decimal between 1 and 2 and was used to quantify damage complexity. A larger fractal dimension indicates a more complex crack distribution and more severe damage. The calculation of *D_f_* was performed according to the following steps:

1. The original image is subjected to binarization processing to enhance the contrast of cracks. Taking the image processing of U-CF0.75 as an example, the procedure is illustrated in Figure 7.

2. MATLAB R2023a was employed to divide the image into square grids with different side lengths r to cover the crack image. In this study, the selected grid scales were 5, 10, 25, 50, 75 and 100 pixels, respectively. The minimum number of grids N(r) occupied by cracks at each scale r was counted, as shown in Figure 8:

3. The logarithms of different scales r and the corresponding grid numbers *N*(r) occupied by cracks were calculated and plotted in a double logarithmic coordinate system. The least squares method was used to fit the data points, as illustrated in Figure 9. If the *R*^2^ of the fitting result is greater than 0.95, the slope of the fitted function is determined as the fractal dimension *D_f_* of the crack image. The fractal dimension is calculated by Equation (9):

(8) $$\ln N\left(r\right)=-D\ln r+C$$

(9) $$D_{f}=-\underset{r\to 0}{\lim}\frac{\ln N\left(r\right)}{\ln r}$$

Following the above procedure, *r* values of 5, 10, 25, 50, 75, and 100 were selected, and the corresponding numbers of grids *N*(r) occupied by cracks were counted. According to the fitting results, the coefficient of determination *R*^2^ for all test groups is greater than 0.95, indicating that crack propagation in fiber-reinforced concrete under impact loading exhibits favorable self-similarity and is suitable for damage analysis using fractal theory.

## 3. Test Results and Discussion

### 3.1. Compressive and Tensile Strength

**Figure 10:**
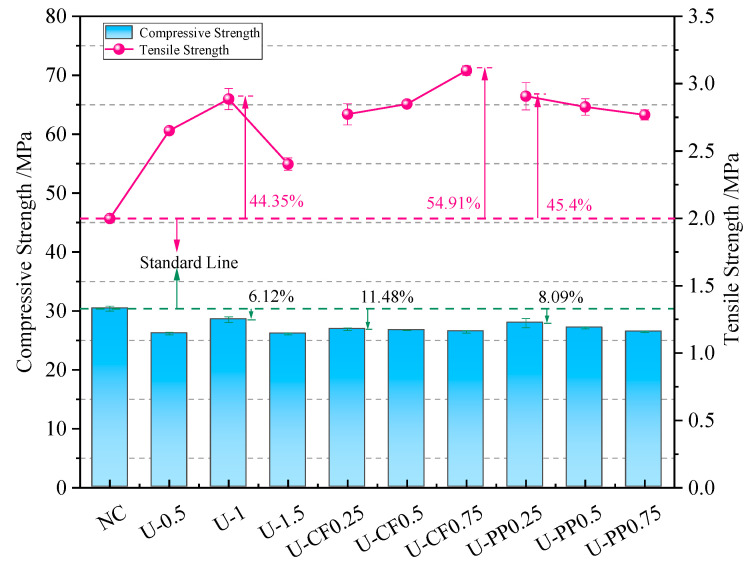
Results of compressive strength and splitting tensile strength of concrete.

Table 3 presents the test results of the mechanical properties of each group. The cube compressive strength results are shown in Figure 10. It can be observed that the compressive strength of concrete decreases after fiber incorporation. The compressive strength of specimens with single UHMWPE fiber is slightly lower than that of plain concrete without fiber, and it first increases and then decreases with increasing fiber content. The optimal volume fraction of UHMWPE fiber is 1%, with a compressive strength reduction of only 6.13% compared with plain concrete. When the UHMWPE fiber content is kept constant at 1%, the strength reduction rates corresponding to CF volume fractions of 0.25%, 0.5%, and 0.75% are 11.48%, 12.14%, and 12.89%, respectively; while those corresponding to PP volume fractions of 0.25%, 0.5%, and 0.75% are 8.12%, 10.82%, and 12.96%, respectively. Compared with the hybridization of two high-modulus fibers, the hybridization of high-modulus and low-modulus fibers causes less reduction in compressive strength. Low-modulus fibers can inhibit the initiation and propagation of microcracks inside the matrix during curing, thereby improving the compactness of concrete. After concrete cracking under compression, high-modulus fibers at the crack surfaces provide a bridging effect to restrain crack propagation and maintain the integrity of concrete. The two types of fibers complement each other, improving toughness without significantly reducing compressive strength. In contrast, for concrete hybridized with two high-modulus fibers, the cracking stress is insufficient to cause fiber fracture, so the fibers cannot fully exert their reinforcing effect.

The splitting tensile strength of concrete with single UHMWPE fiber shows a trend of first increasing and then decreasing. The optimal volume fraction of UHMWPE fiber is 1%, and its splitting tensile strength is 44.2% higher than that of plain concrete. When the UHMWPE fiber content exceeds 1%, the splitting tensile strength begins to decrease. This is because excessive incorporation of a single fiber type introduces more defects inside the concrete, thereby reducing its strength. For hybrid fiber concrete containing UHMWPE fiber and carbon fiber (CF), the splitting tensile strength increases continuously with increasing CF volume fraction. When the CF volume fraction increases from 0.25% to 0.75%, the splitting tensile strength of the FRC increases by 38.7%, 41.2%, and 53.2%, respectively.

For hybrid fiber concrete containing UHMWPE fiber and polypropylene (PP) fiber, the splitting tensile strength decreases continuously with increasing PP fiber content, but the rate of change is not obvious. When the PP fiber volume fraction increases from 0.25% to 0.75%, the splitting tensile strength of the FRC increases by 42.7%, 40.2%, and 39.2%, respectively. Compared with concrete containing only UHMWPE fiber, the splitting tensile strength of hybrid fiber concrete increases when the total volume fraction of the two fibers exceeds 1%. Compared with U-1.5 concrete, the splitting tensile strength of U-CF0.5, U-CF0.75, U-PP0.5, and U-PP0.75 increases by 11.5%, 21.1%, 10.7%, and 9.9%, respectively. Both hybrid methods compensate to some extent for the defects generated inside the concrete matrix by excessive single-fiber incorporation, allowing the fibers to exert their respective reinforcing effects.

The combined results of cube compressive strength and splitting tensile strength indicate that fiber hybridization with different elastic moduli has a significant influence on the mechanical properties of concrete. Compared with the U-PP system, the U-CF system exhibited slightly lower compressive strength while still maintaining satisfactory toughening performance. This behavior can be attributed to the ability of low-modulus fibers to suppress the initiation and propagation of microcracks during curing, thereby improving the compactness of the concrete matrix. In contrast, high-modulus fibers mainly act as crack-bridging components after cracking, delaying crack propagation and enhancing the synergistic effect between fibers. The splitting tensile results further demonstrate that increasing carbon fiber content enhances the crack-bridging capacity and restrains lateral deformation, leading to improved tensile resistance.

### 3.2. Flexure Test Result

**Figure 11:**
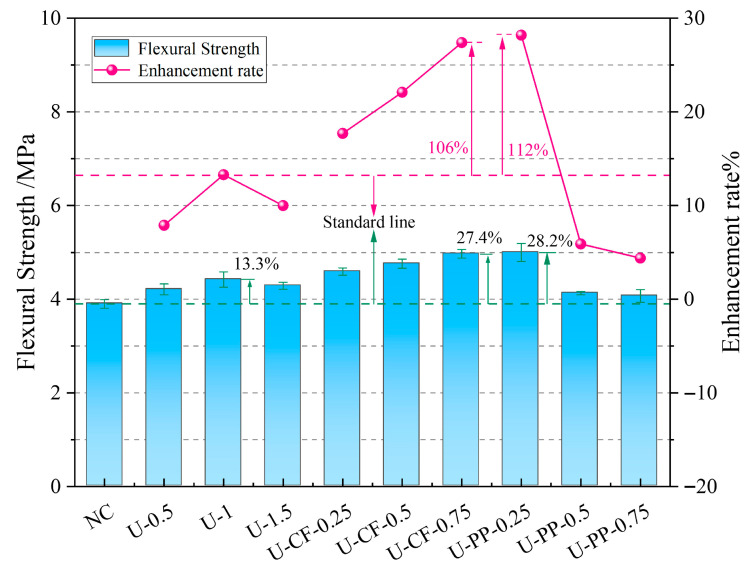
Flexural strength results for each test group.

As shown in Figure 11 and Table 3, the flexural strength of concrete specimens reinforced with UHMWPE fiber alone first increases and then decreases with increasing fiber volume fraction. The optimal performance is achieved at a UHMWPE fiber volume fraction of 1%, corresponding to a 13% improvement in flexural strength compared with plain concrete without fiber addition. For fiber-reinforced concrete hybridized with UHMWPE fiber and carbon fiber (CF), the flexural strength increases continuously with increasing CF volume fraction. No declining trend is observed even when the total fiber volume fraction reaches or exceeds 1.5%, with improvements of 8% and 12.4%, respectively. When the CF volume fraction rises from 0.25% to 0.75%, the flexural strength increases by 17.7%, 22.1%, and 27.4%, respectively.

In contrast, for concrete hybridized with UHMWPE fiber and polypropylene (PP) fiber, the flexural strength decreases continuously with increasing PP fiber volume fraction. The maximum flexural strength is obtained with 0.25% PP fiber added on the basis of 1% UHMWPE fiber, showing a 28.2% increase relative to plain concrete. This indicates that fiber incorporation enhances the tensile strength of the tension zone in prismatic specimens, thereby improving flexural strength. After cracking in the tensile zone at the bottom of the concrete, the bridging effect of fibers across the crack surfaces [20] restrains the two sides of the crack, disperses stress, and transfers vertical loads to adjacent concrete, enabling monolithic force transfer and increasing load-bearing capacity.

The flexural test results of the U-CF and U-PP hybrid fiber-reinforced concretes indicate that the PP fiber system exhibited the best performance at a fiber volume fraction of 0.25 vol%, where low-modulus PP fibers most effectively restrained early microcrack development. Further increases in PP content led to a gradual and pronounced reduction in flexural strength, primarily due to fiber agglomeration and the resulting deterioration in dispersibility. In contrast, the flexural strength of the CF-containing mixtures increased continuously with increasing fiber content and reached its optimum at 0.75 vol%, demonstrating that the crack-bridging effect of high-modulus fibers plays a more significant role in enhancing flexural load-bearing capacity.

### 3.3. Impact Test Result

#### 3.3.1. The Development Process of Impact Cracks

**Figure 12:**
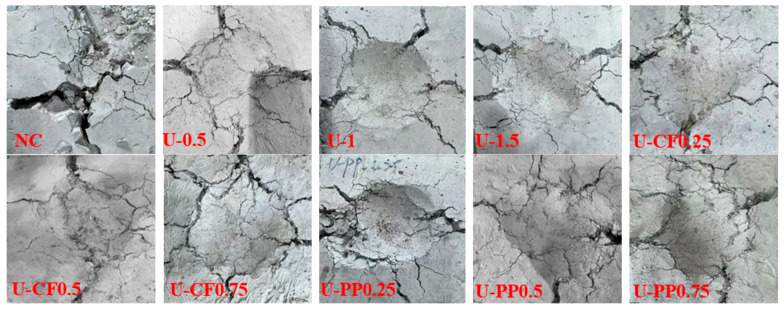
The process of impact cracking on the top of specimens.

The failure processes of each test group are illustrated in Figure 12. Under vertical impact loading, plain concrete without fiber addition exhibits an obvious brittle failure mode. When the impactor contacts the specimen, cracks initiate instantaneously. As impact repetitions increase, distinct major cracks appear on the concrete surface and gradually propagate and extend. Eventually, severe separation and spalling are observed at specimen failure. UHMWPE fiber-reinforced concrete (UFRC) maintains good integrity after impact failure, without the spalling or separation seen in plain concrete. Furthermore, as the fiber volume fraction increases, the number of surface cracks increases while the width of several major cracks at the top surface decreases, showing a propagation pattern that extends radially from the impact point to the surrounding area.

Similarly to singly doped UHMWPE fiber concrete, U-CFHFRC exhibits no obvious concrete spalling during impact failure. With increasing carbon fiber volume fraction, the number of major cracks on the specimen surface increases from 2 to 7, and the width of the major cracks at the top surface decreases at failure. In contrast, U-PPHFRC still retains the integrity characteristic of fiber-reinforced concrete at failure. With increasing polypropylene fiber volume fraction, the number of major cracks on the specimen top surface remains almost unchanged, staying at 2 to 3, whereas the number of secondary cracks increases significantly with fiber content.

#### 3.3.2. The Effect of Fiber Hybrid Methods on Impact Resistance

The results of the drop-weight impact tests are summarized in Table 4. The number of impacts at the initiation of the first crack (*N*_1_), the number of impacts at failure (*N*_2_), the initial cracking impact energy (*W*_1_), the failure impact energy (*W*_2_), the post-cracking energy absorption, and the ductility ratio (*β*) are all presented in Table 4.

**Figure 13:**
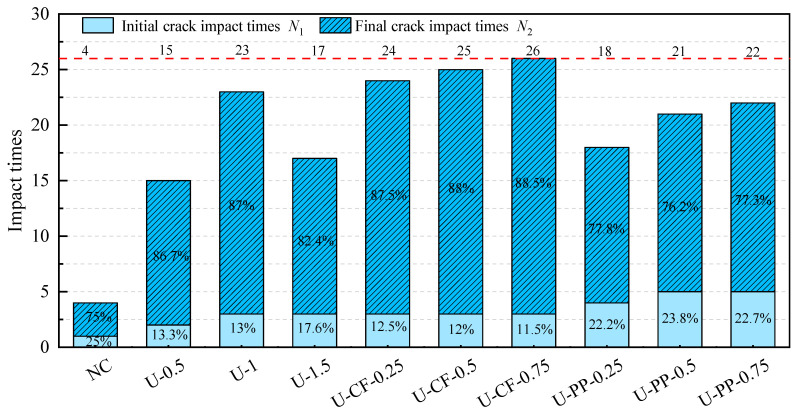
The number of impacts of concrete.

As observed from Table 4 and Figure 13, the difference between *N*_1_ and *N*_2_ for plain concrete specimens without fiber reinforcement is minimal, indicating that crack initiation and ultimate failure occur almost simultaneously under impact loading and reflect pronounced brittle behavior. Compared with plain concrete without fibers, the incorporation of any type of fiber significantly enhances impact resistance, as evidenced by the increased number of impacts sustained. Fibers are randomly dispersed within the concrete matrix, forming numerous micro-reinforcements. These fibers effectively inhibit the continuous propagation of cracks induced by impact loading, thereby imparting superior impact resistance to the concrete [24].

For UFRC, with increasing fiber volume fraction, both the number of impacts and the impact strength of the concrete exhibit a trend of first increasing and then decreasing. As the fiber volume fraction increases from 0.5% to 1.5%, the number of impacts at initial cracking increases by 100%, 200%, and 200%, respectively; the number of impacts at failure increases by 333.3%, 566.7%, and 366.7%, respectively; and the impact strength increases by 300%, 510%, and 330%, respectively. Notably, when the UHMWPE fiber content is 1%, the impact resistance and impact strength of the concrete reach optimal values. For U-CFHFRC, as the carbon fiber volume fraction increases, both the number of impacts and the impact strength continuously improve. Compared with ordinary fiber-reinforced concrete, increasing the carbon fiber content from 0.25% to 0.75% has little influence on the initial cracking impact number, but significantly improves the failure impact number and impact strength. Specifically, the failure impact number increases by 600%, 633.3%, and 666.7%, while the impact strength increases by 330%, 660%, and 1150%, respectively.

For U-PPHFRC, compared with ordinary concrete, the number of impacts at initial cracking, the number of impacts at failure, and the impact strength all increase with increasing polypropylene fiber volume fraction. When the polypropylene fiber volume fraction increases from 0.25% to 0.75%, the number of impacts at initial cracking increases by 300%, 400%, and 400%, respectively, while the number of impacts at failure increases by 366.7%, 433.3%, and 466.7%, respectively. This indicates that the incorporation of polypropylene fibers significantly enhances impact resistance in the early stage (before cracking), whereas its improvement in the later stage (post-cracking) is less pronounced [25].

**Figure 14:**
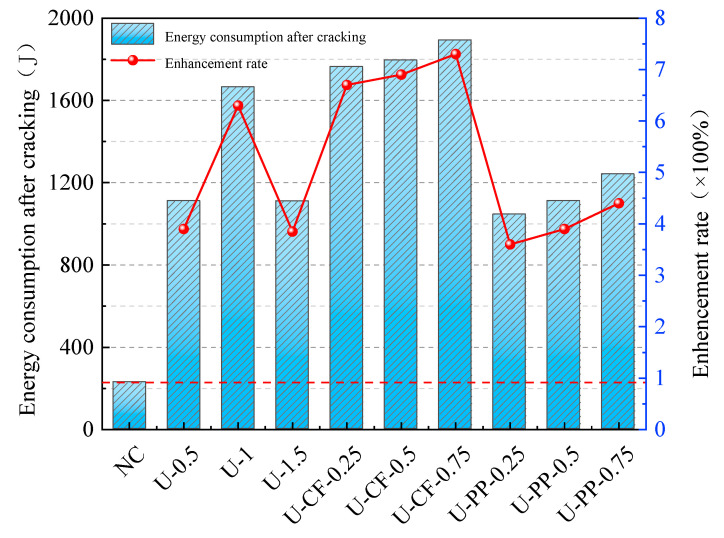
Energy consumption after concrete cracking resistance.

Post-cracking energy absorption reflects the ability of fiber-reinforced concrete to continue absorbing impact energy after cracking under impact loading, and it is one of the key indicators for evaluating material impact toughness [26]. Figure 14 presents the post-cracking energy absorption of all specimens. As shown in the figure, plain concrete without fibers exhibits a very limited capacity for energy absorption after cracking, demonstrating a typical brittle failure mode characterized by failure upon cracking. In contrast, concrete incorporating fibers shows a significantly enhanced ability to absorb energy after cracking under impact loading, indicating that the inclusion of fibers effectively improves the impact toughness of concrete [27].

For concrete reinforced solely with UHMWPE fibers, the post-cracking energy absorption first increases and then decreases with increasing fiber volume fraction, with an optimal fiber content of 1%. Excessive fiber addition may lead to fiber agglomeration within the concrete matrix, resulting in internal defects. During impact loading, cracks tend to initiate from these defects, thereby reducing impact resistance. Based on a constant incorporation of 1 vol% UHMWPE fibers, the addition of carbon fibers at different volume fractions leads to a continuous increase in the post-cracking energy absorption of hybrid fiber-reinforced concrete. This indicates that carbon fibers significantly enhance the energy absorption capacity of concrete after cracking. Moreover, this hybrid fiber system overcomes the limitation associated with the deterioration of impact resistance caused by excessive incorporation of a single fiber type. When PP fibers are added at different volume fractions on the basis of 1 vol% UHMWPE fibers, the post-cracking energy absorption increases with increasing PP content; however, the overall values remain lower than those of concrete reinforced with 1 vol% UHMWPE fibers alone. Although U-PPHFRC exhibits better post-cracking energy absorption than ordinary concrete, PP fibers do not significantly enhance the post-cracking energy absorption compared with UFRC.

To evaluate the impact toughness of hybrid fiber-reinforced concrete, this study introduces a quantitative indicator termed the hybrid effect index. This index characterizes the synergistic or antagonistic effects of different fiber combinations by comparing performance differences between hybrid fiber-reinforced concrete and single-fiber-reinforced concrete [19]. The index is calculated based on experimental data. By analyzing the nonlinear relationship between performance enhancement and fiber parameters such as volume fraction and elastic modulus, a mathematical expression is established. The formula for calculating the hybrid effect index is given as follows:

(10) $$\alpha_{R}=\frac{R_{H}-R_{0}}{\left(R_{i}-R_{0}\right)\beta_{i}}$$

(11) $$\beta_{i}=\frac{V_{i}}{V}$$

(12) $$\sum \beta_{i}=1$$

where *η* denotes the hybrid effect index; *R_H_* is the impact toughness of hybrid fiber-reinforced concrete; *R*_0_ represents the impact toughness of plain concrete; and *R*_i_ denotes the impact toughness of concrete reinforced with a single type of ultra-high-molecular-weight polyethylene (UHMWPE) fiber. $\beta_{i}$ is the volume fraction ratio of UHMWPE, carbon fiber (CF), and polypropylene (PP) fibers; *V*i is the volume fraction of UHMWPE/CF/PP fibers; and *V* is the total fiber volume fraction in the hybrid fiber-reinforced concrete. When $\alpha_{R}$ > 1, the results suggest a possible synergistic effect within the concrete matrix; when $\alpha_{R}$ < 1, they exhibit an antagonistic effect.

**Figure 15:**
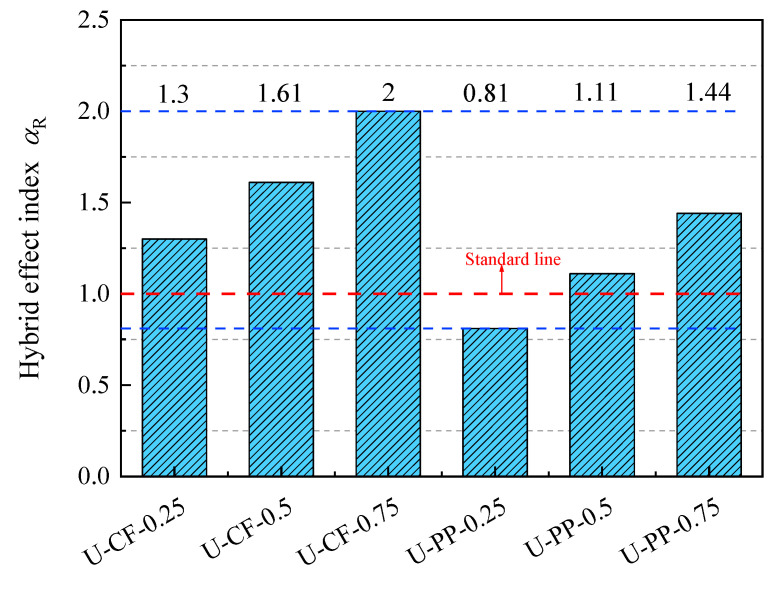
Hybrid effect index of impact toughness.

As shown in Figure 15, the hybrid effect indices for both crack initiation and failure of concrete incorporating the UHMWPE–CF combination are generally greater than 1. Moreover, the hybrid effect index at failure increases with the increasing volume fraction of carbon fibers, indicating that the combination of two high elastic modulus fibers exhibits a pronounced synergistic effect under impact loading. Throughout the impact process from crack initiation to ultimate failure, an effective “bridging effect” is mobilized, thereby enhancing the crack resistance and impact performance of the concrete. This behavior can be attributed to the high tensile strength of high-modulus fibers. After cracking, the fibers across the crack surfaces are less prone to rupture and can continue to bridge the cracks. In addition, as the fiber volume fraction increases, the spacing between fibers decreases and the number of fibers intersecting the crack plane increases, further improving the crack resistance of the concrete. In contrast, for the UHMWPE–PP fiber combination, the hybrid effect index is generally lower. In particular, the hybrid effect index of specimen U-PP0.25 is less than 1, indicating that, at this volume fraction, the fiber combination produces an antagonistic effect on the impact resistance of the concrete.

### 3.4. Results of Fractal Dimension Analysis

**Figure 16:**
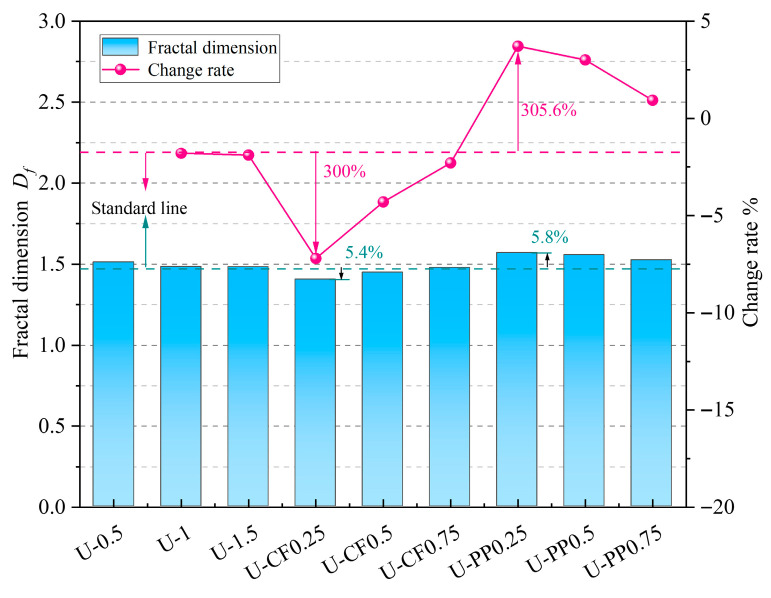
Fractal dimension of each test group.

As illustrated in Figure 16 and Table 5, fractal dimension analysis reveals that the degree of impact-induced damage in fiber-reinforced concrete varies with different fiber combinations. For concrete reinforced solely with UHMWPE fibers, the fractal dimension decreases continuously with increasing fiber volume fraction, indicating that fiber incorporation effectively reduces the impact damage to the specimens. For the U-CFHFRC specimens, the fractal dimension increases with increasing carbon fiber content, but remains overall lower than that of the UHMWPE-only specimens. This indicates that, during impact, high-modulus fibers effectively exert a bridging effect within the concrete matrix, thereby suppressing crack propagation and mitigating impact damage.

In contrast, for U-PPHFRC specimens, the fractal dimension is generally higher than that of the UHMWPE-only specimens. However, it decreases gradually with increasing polypropylene fiber content. This suggests that PP fibers can intensify local cracking and energy dissipation during impact, leading to a more complex crack pattern and, in some cases, greater damage complexity.

**Figure 17:**
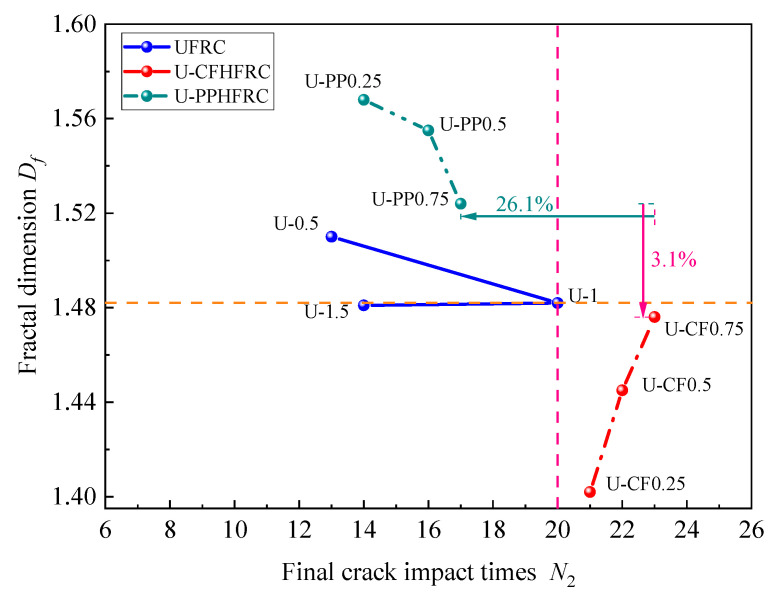
Overall comparison chart of fractal dimension results.

The variation in fractal dimension *D_f_* for concrete under different hybrid fiber combinations is presented in Figure 17. For UFRC, the fractal dimension decreases slowly, with minor overall fluctuations, remaining between 1.48 and 1.52. U-PPHFRC exhibits the highest initial fractal dimension; however, with increasing fiber volume fraction and number of impacts, it shows a pattern of dispersed damage under low-frequency impacts and concentrated damage under high-frequency impacts. In contrast, U-CFHFRC demonstrates the opposite trend: as the fiber volume fraction and impact resistance increase, the fractal dimension steadily rises, indicating more concentrated damage under impact and stronger resistance to cumulative impacts.

Fractal dimension analysis reveals that fibers with different elastic moduli exhibit distinct working mechanisms within the concrete under impact loading. High-modulus fibers suppress crack propagation through a bridging effect and prevent tensile failure of the concrete due to their high tensile strength, thereby enhancing the impact resistance of the concrete. In contrast, low-modulus fibers, such as polypropylene fibers, can inhibit the development of microcracks and improve concrete density during the early curing stage. Under impact loading, these fibers promote controlled cracking through stress dispersion and energy absorption via fiber fracture, thereby improving the impact resistance of the concrete.

### 3.5. Analysis of Impact Resistance of HFRC Based on Weibull Distribution Model

#### 3.5.1. Preliminary Weibull Statistical Analysis of HFRC Impact Resistance

In the study of the impact resistance of concrete materials, the Weibull distribution function has become an important tool for analyzing the impact life and damage of concrete due to its unique mathematical properties and physical applicability [28,29,30,31]. In the present study, a two-parameter Weibull distribution model was adopted to preliminarily evaluate the statistical characteristics of the impact resistance results of hybrid fiber-reinforced concrete (HFRC). Assuming that the impact life *N* of fiber-reinforced concrete follows a Weibull distribution, the cumulative distribution function can be expressed as:

(13) $$f\left(N\right)=\frac{b}{N_{a}}{\left(\frac{N}{N_{a}}\right)}^{b-1}\exp\left[-{\left(\frac{N}{N_{a}}\right)}^{b}\right]\begin{array}{cc} & \left(\infty >N\geq N_{0}\right) \end{array}$$

where *b* is the shape parameter, *N*_0_ is the minimum life parameter, and *N_a_* is the characteristic life parameter, with *b* > 0 and *N_a_* > *N*_0_. Considering the safety and reliability of the specimens during service, the minimum life parameter *N*_0_ is set to zero.

The reliability function *P*_2_ is expressed as shown in Equation (14):

(14) $$P_{2}=1-P_{1}=\exp\left[-{\left(\frac{N}{N_{a}}\right)}^{b}\right]$$

By taking the natural logarithm twice on both sides of Equation (14), Equation (15) is obtained:

(15) $$\ln\left[\ln\left(\frac{1}{P_{2}}\right)\right]=b\ln\left(\frac{1}{N_{a}}\right)+b\ln\left(N\right)$$

By substituting *Y* = ln[ln(1/*P*_2_)], *X* = ln(*N*), and *a* = *b*ln(1/*N_a_*) into Equation (18), the relationship *Y* = *a* + *bX* is obtained. The parameters *a*, *b*, and *R*^2^ are determined through linear regression analysis. To verify whether the impact life of the specimens follows a Weibull distribution, a good linear correlation should exist between ln[ln(1/*P*_2_)] and ln(*N*). The reliability *P*_2_ must first be calculated, with the calculation method given by Equation (16):

(16) $$P_{2}=1-\frac{i}{m+1}$$

where *m* denotes the total number of specimens in each test group, which is 3 in the present study, and *i* represents the rank order of the impact resistance results for the specimens in each group, arranged from smallest to largest.

**Figure 18:**
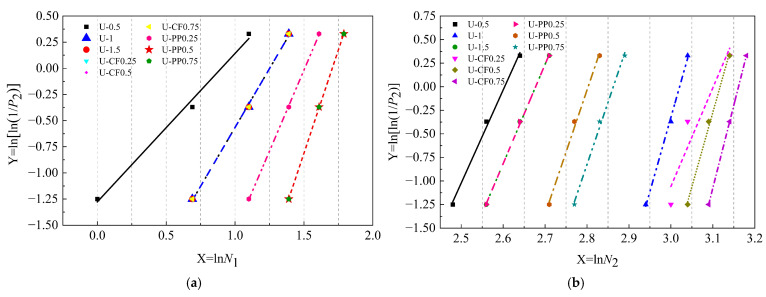
Linear regression curves of *N*_1_ and *N*_2_ in Weibull distribution: (**a**) Initial-crack impact times *N*_1_; (**b**) final-crack impact times *N*_2_.

As shown in Figure 18 and Table 6, the correlation coefficients *R*^2^ for all test groups are greater than 0.9, indicating a significant linear relationship between *X* = ln*N* and *Y* = ln[ln(1/*P*_2_)]. This confirms that the initial cracking number of impacts *N*_1_ and the failure number of impacts *N*_2_ for all concrete specimens in this study follow a Weibull distribution. These results demonstrate that the two-parameter Weibull distribution function can reasonably describe the impact life of fiber-reinforced concrete [32]. The Weibull analysis presented herein should be regarded as a preliminary statistical evaluation rather than a rigorous reliability assessment.

#### 3.5.2. HFRC Impact Damage Analysis

In engineering materials subjected to normal service loads, the initiation, evolution, and propagation of internal defects ultimately lead to material failure. The extent of damage can be quantitatively characterized by a damage variable, denoted as *D*. Assuming that the damage strength after n impact events follows a Weibull distribution, the corresponding damage variable *D* can also be described by a Weibull distribution. The mathematical expression is given as follows:

(17) $$f\left(n\right)=\frac{\beta }{\eta }{\left(\frac{n}{\eta }\right)}^{\beta -1}\exp\left[-{\left(\frac{n}{\eta }\right)}^{\beta }\right]$$

From the perspective of impact-induced damage in fiber-reinforced concrete, it can be observed that the degree of damage progressively intensifies with the number of impact load applications. It can therefore be reasonably assumed that, during the drop-weight impact process, the damage evolution in concrete and the failure probability develop simultaneously. After *n* impact events, the failure probability of concrete is denoted as *P*(*n*), and the corresponding damage variable is defined as *D*(*n*). When impact failure occurs, both the failure probability and the damage variable reach unity, i.e., *P*(*n*) *=* 1 and *D*(*n*) *=* 1. Accordingly, the impact damage model of concrete based on the Weibull distribution can be expressed as follows:

(18) $$D\left(n\right)=1-\exp\left[-{\left(\frac{n}{\eta }\right)}^{\beta }\right]$$

In the above formulation, n denotes the number of impact events, while the parameters *β* and *η* can be determined by combining Equations (13)–(17) with the data presented in Table 6. Substituting the calculated parameters into Equation (18), the impact damage evolution equations for each experimental group can be obtained, as summarized in Table 7.

**Figure 19:**
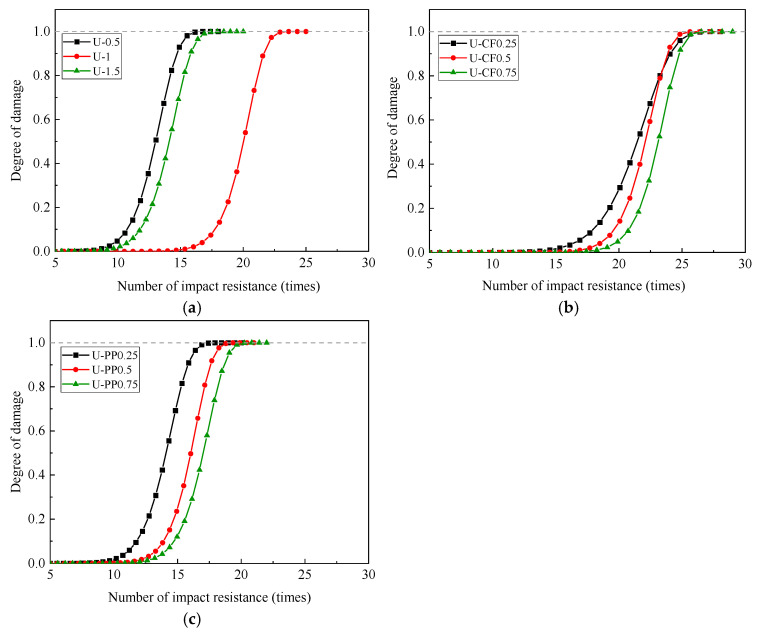
Impact damage evolution diagram of fiber-reinforced concrete. (**a**) Impact damage evolution diagram of UFRC; (**b**) impact damage evolution diagram of U-CFHFRC; (**c**) impact damage evolution diagram of U-PPHFRC.

Based on the impact damage evolution equations presented in Table 7, the corresponding damage evolution curves for each specimen group are plotted, as shown in Figure 19. It can be observed that the damage evolution of fiber-reinforced concrete under repeated impact loading exhibits a clear progressive trend. In the initial stage of impact, the damage level remains relatively low, and the propagation of internal microcracks and pores is slow. As the number of impact events increases, cracks within the matrix rapidly initiate and propagate, leading to a significant increase in impact-induced damage. When *D*(*n*) = 1, the concrete is considered to have reached failure.

As illustrated in Figure 19, the results obtained from the proposed impact damage evolution model show good agreement with the experimental findings analyzed using fractal theory. This consistency indicates that the model can effectively capture the damage growth process under impact loading.

## 4. Conclusions

In this study, static tests and drop-weight impact tests were conducted to investigate the mechanical properties and impact resistance of concrete incorporating hybrid fibers with multiple elastic moduli. Based on the experimental results and predictive analysis, the main conclusions are summarized as follows:

1. Hybrid fiber-reinforced concrete exhibits greater mechanical enhancement than single-fiber concrete, with UHMWPE/CF-HFRC showing the best static performance. In addition, fiber incorporation prevents spalling at failure, maintains structural integrity, and improves ductility.
2. Fibers with different elastic moduli act at different loading stages. In the early impact stage, low-modulus PP fibers inhibit microcrack growth, while in the later stage, UHMWPE and CF exhibit a synergistic bridging effect after cracking.
3. UHMWPE/PP-HFRC shows a higher fractal dimension than UHMWPE/CF-HFRC, indicating that low-modulus fibers absorb impact energy by promoting cracking, while high-modulus fibers better restrain crack growth and enhance impact resistance via bridging.
4. PP fibers mainly enhanced the crack initiation resistance of concrete, while CFs provided superior crack-bridging and post-cracking energy absorption capacity due to their higher elastic modulus. Among them, U-CF0.75 not only increased the impact life but also reduced the impact damage.
5. The two-parameter Weibull distribution effectively characterizes the impact resistance of fiber-reinforced concrete, with predicted impact life consistent with test results. The proposed damage evolution equation also captures the impact damage process well.

## Figures and Tables

**Table 1:** Material properties of fiber.

Fibers	Length (mm)	Diameter (μm)	Aspect Ratio	Density (g/cm^3^)	Tensile Strength (MPa)	Modulus of Elasticity (GPa)	Fracture Elongation (%)
CF	10	7	1429	1.75	4900	230	2
UHMWPE	9	25	360	0.97	3100	122	3.5
PP	9	18	500	0.91	500	3.9	10–28

**Table 2:** Mixture proportions and test groups.

Groups	Volume Ratio (%)			Mixture Proportions (Unit Weight kg/m^3^)				
	CF	UHMWPE	PP	Cement	Gravel	Water	Silica Sand	Water-Reducing Admixture
NC	0	0	0	375	1276.45	161.25	687.3	2.25
U-0.5	0	0.5	0					
U-1	0	1	0					
U-1.5	0	1.5	0					
U-CF0.25	0.25	1	0					
U-CF0.5	0.5	1	0					
U-CF0.75	0.75	1	0					
U-PP0.25	0	1	0.25					
U-PP0.5	0	1	0.5					
U-PP0.75	0	1	0.75					

**Table 3:** Test results of mechanical properties for each group.

Group	Compressive Strength (MPa)				Tensile Strength (MPa)				Flexural Strength (MPa)			
	1	2	3	Mean ± SD	1	2	3	Mean ± SD	1	2	3	Mean ± SD
NC	30.72	29.91	30.55	30.39 ± 0.35	1.99	1.97	2.01	1.99 ± 0.02	4.00	3.93	4.11	4.01 ± 0.07
U-0.5	26.29	26.31	25.96	26.17 ± 0.16	2.66	2.67	2.71	2.68 ± 0.02	4.14	4.14	4.34	4.21 ± 0.09
U-1	28.22	29.12	28.25	28.53 ± 0.42	2.96	2.82	2.83	2.87 ± 0.06	4.54	4.48	4.23	4.42 ± 0.13
U-1.5	26.23	26.00	26.02	26.1 ± 0.10	2.57	2.48	2.51	2.52 ± 0.04	4.22	4.29	4.37	4.29 ± 0.06
U-CF0.25	27.14	26.89	26.67	26.9 ± 0.19	2.71	2.72	2.85	2.76 ± 0.06	4.67	4.58	4.51	4.59 ± 0.07
U-CF0.5	26.67	26.68	26.7	26.7 ± 0.01	2.82	2.82	2.78	2.81 ± 0.02	4.65	4.8	4.83	4.76 ± 0.08
U-CF0.75	26.75	26.32	26.36	26.47 ± 0.19	3.08	3.06	3.01	3.05 ± 0.03	5.05	4.87	4.98	4.97 ± 0.07
U-PP0.25	27.27	27.75	28.77	27.93 ± 0.63	2.78	2.79	2.96	2.84 ± 0.08	5.19	4.81	5.00	5.00 ± 0.16
U-PP0.5	26.97	27.23	26.99	27.1 ± 0.12	2.86	2.74	2.78	2.79 ± 0.05	4.17	4.13	4.10	4.13 ± 0.03
U-PP0.75	26.34	26.54	26.47	26.45 ± 0.08	2.75	2.81	2.74	2.77 ± 0.03	3.92	4.06	4.19	4.06 ± 0.11

**Table 4:** The impact resistance test result of HFRC.

Specimens	*N*_1_				*N*_2_				Initial-Crack Impact Energy:*W*_1_ (J)	Final-Crack Impact Energy:*W*_2_ (J)	Δ*W* (J)	Ductility Ratio:*β*
	1	2	3	Avg.	1	2	3	Avg.				
NC	1	1	1	1	3	3	4	3	98	294	196	2
U-0.5	2	2	2	2	14	13	13	13	196	1274	1078	5.5
U-1	4	3	3	3	20	21	20	20	294	1960	1666	5.67
U-1.5	3	3	3	3	14	14	15	14	294	1372	1078	3.67
U-CF0.25	2	4	3	3	20	20	23	21	294	2058	1764	6
U-CF0.5	4	3	3	3	20	22	23	22	294	2156	1862	6.33
U-CF0.75	3	4	3	3	22	23	23	23	294	2254	1960	6.67
U-PP0.25	5	3	4	4	15	14	14	14	392	1372	980	2.5
U-PP0.5	6	5	4	5	15	17	16	16	490	1568	1078	2.2
U-PP0.75	5	6	4	5	16	18	18	17	490	1666	1176	2.4

**Table 5:** Summary table of fractal dimension calculation results.

Group	U-0.5	U-1	U-1.5	U-CF0.25	U-CF0.5	U-CF0.75	U-PP0.25	U-PP0.5	U-PP0.75
*D_f_*	1.510	1.482	1.481	1.402	1.445	1.476	1.568	1.555	1.524

**Table 6:** Regression parameter values.

Specimen	*N*_1_			*N*_2_		
	*b*	*a* = *b*ln(*N*_a_)	*R* ^2^	*b*	*a* = *b*ln(*N*_a_)	*R* ^2^
U-0.5	1.42	1.28	0.993	9.875	25.71	0.996
U-1	2.25	2.81	0.998	15.71	47.46	0.997
U-1.5	2.25	2.81	0.998	10.54	28.23	0.999
U-CF0.25	2.25	2.81	0.998	10.46	32.44	0.907
U-CF0.5	2.25	2.81	0.998	15.80	49.25	0.996
U-CF0.75	2.25	2.81	0.998	17.56	55.50	0.999
U-PP0.25	3.09	4.66	0.999	10.54	28.23	0.999
U-PP0.5	3.95	6.74	0.999	13.17	36.90	0.996
U-PP0.75	3.95	6.74	0.999	13.17	37.69	0.996

**Table 7:** Summary of impact evolution equations for fiber-reinforced concrete.

$\text{U-0.5:}D\left(n\right)=1\;-\;\exp\left[-{\left(\frac{n}{13.5}\right)}^{9.88}\right]$	$\text{U-CF}0.75:D\left(n\right)=1\;-\;\exp\left[-{\left(\frac{n}{23.6}\right)}^{17.56}\right]$
$\text{U-1}:D\left(n\right)=1\;-\;\exp\left[-{\left(\frac{n}{20.5}\right)}^{15.71}\right]$	$\text{U-PP}0.25:D\left(n\right)=1\;-\;\exp\left[-{\left(\frac{n}{14.6}\right)}^{10.54}\right]$
$\text{U-1.5}:D\left(n\right)=1\;-\;\exp\left[-{\left(\frac{n}{14.6}\right)}^{10.54}\right]$	$\text{U-PP}0.5:D\left(n\right)=1\;-\;\exp\left[-{\left(\frac{n}{16.5}\right)}^{13.17}\right]$
$\text{U-CF}0.25:D\left(n\right)=1\;-\;\exp\left[-{\left(\frac{n}{22.2}\right)}^{10.46}\right]$	$\text{U-PP}0.75:D\left(n\right)=1\;-\;\exp\left[-{\left(\frac{n}{17.5}\right)}^{13.17}\right]$
$\text{U-CF}0.5:D\left(n\right)=1\;-\;\exp\left[-{\left(\frac{n}{22.6}\right)}^{15.8}\right]$	

## Data Availability

The original contributions presented in this study are included in the article. Further inquiries can be directed to the corresponding author.
